# Impact of Incretin Hormone Receptors on Insulin-Independent Glucose Disposal in Model Experiments in Mice

**DOI:** 10.3389/fendo.2021.680153

**Published:** 2021-06-08

**Authors:** Tina Ovlund, Giovanni Pacini, Bo Ahrén

**Affiliations:** ^1^ Department of Clinical Sciences Lund, Lund University, Lund, Sweden; ^2^ Independent Researcher, Padova, Italy

**Keywords:** GLP-1, GIP, glucose disposal, insulin-independent, diazoxide, fluafent

## Abstract

A large contribution to glucose elimination from the circulation is achieved by insulin-independent processes. We have previously shown that the two incretin hormones, glucose-dependent insulinotropic polypeptide (GIP) and glucagon-like peptide-1 (GLP-1) increase this process and, therefore, seem to contribute to glucose disposal both through this effect and through the classical incretin effect resulting in enhanced insulin levels. We have now explored in more detail the potential contribution by incretin hormone receptors to insulin-independent processes for glucose elimination. To that end, we have performed intravenous glucose tests (0.35g/kg) in C57BL/6J mice and analyzed glucose elimination rate and glucose effectiveness (i.e., insulin-independent glucose disposal, S_G_) in wildtype mice and in mice with genetic deletion of GIP receptors or GLP-1 receptors. We performed studies with or without complete blockade of insulin secretion by the drug diazoxide (25 mg/kg). The mice were anesthetized with a novel fentanyl citrate/fluanisone formulation, called Fluafent, together with midazolam. Initially we demonstrated that glucose and insulin data after intravenous and oral glucose were not different using this anesthesia compared to the previously commonly used combination of Hypnorm^R^ and midazolam. The results show that S_G_ was reduced in GLP-1 receptor knockout mice, whereas there was no difference between GIP receptor knockout mice and wildtype mice, and this was evident both under normal conditions and after complete inhibition of insulin secretion. The study therefore indicates that insulin-independent glucose elimination requires active GLP-1 receptors and thus that the two incretin hormone receptor types show dissociated relevance for this process.

## Introduction

An important effect of insulin is to stimulate the transportation of glucose into insulin-sensitive tissue, such as skeletal muscle, and thereby reduce the circulating level of glucose (1). However, glucose disposal may also be regulated by insulin-independent mechanisms, as was initially reported in 1937 (2). This was later confirmed in 1979 by minimal modeling of glucose and insulin data from an intravenous glucose tolerance test (3). Several other studies have also reported that insulin-independent glucose elimination contributes to the removal of glucose from the circulation (4–7). In a recent review the several studies in this were summarized and it was reported that several factors, such as incretin hormones, glucagon and insulin sensitivity all may contribute to insulin-independent glucose disposal (6). The process of insulin-independent glucose disposal is also called glucose effectiveness and can be assessed by minimal model of data achieved from the intravenous glucose tolerance test (2, 4, 6, 8–10). Alternatively, it can also be estimated after inhibiting suprabasal dynamic insulin secretion after intravenous glucose when the resulting glucose disposal is by definition insulin-independent (10). As recently reviewed, insulin-independent glucose disposal is important and contributes by more than 50-70% to glucose disposal in humans and experimental animals (5–7). Furthermore, this process is reduced in obesity and diabetes and may therefore contribute to dysregulation of glucose in these conditions (11–13). It has also been demonstrated that exogenous administration of the incretin hormone glucagon-like peptide-1 (GLP-1) increases glucose effectiveness in human (14) and that both GLP-1 and the other incretin hormone glucose-dependent insulinotropic polypeptide (GIP) increase glucose effectiveness in model experiments in mice (15). This would suggest that the well-known glucose-reducing effects of the two incretin hormones may be mediated both by insulin-dependent (through increase in circulating insulin) and insulin-independent mechanisms. However, whether this implies that glucose effectiveness per se is dependent on activation of GIP or GLP-1 receptors, which both are members of the G protein coupled receptors (GPCRs) superfamily (16, 17), is not known.

To study the potential requirement of active incretin hormone receptors on physiological processes in detail, mice with genetic deletion of GIP or GLP-1 receptors have been generated (18). In this study, we have explored glucose effectiveness in these mice both by assessing glucose effectiveness during an intravenous glucose tolerance test (IVGTT) and by analyzing glucose disposal during an IVGTT when insulin secretion is prevented by diazoxide, which is a drug that potently inhibits insulin secretion (19).

The study was undertaken in anesthetized mice. We have previously used neurolept anesthesia in mice using the conventional fentanyl citrate/fluanisone combination called Hypnorm^R^ (6, 8–10, 15, 20, 21). Since there is a need for an alternative to this formulation, we have developed a novel combination of fentanyl citrate and fluanisone to be used together with midazolam as neurolept anesthesia. This study is the first project in which this novel neurolept anesthesia formulation is used and, therefore, validation of this novel neurolept anesthesia formulation is also reported.

## Methods

### Animals

The generation of GLP-1 receptor knockout mice and GIP receptor knockout mice has been described previously (22). Briefly, mice on a C57BL6J background being heterozygous for the deletion of both the *Glp1r* and *Gipr* genes were generated from double homozygous deletion mutant (DIRKO) mice by rederivation at Taconic Europe (Silkeborg, Denmark). Heterozygotes were mated to yield GLP-1 receptor knockout mice, GIP receptor knockout mice, and wildtype mice. The resulting offspring was used to establish breeding pairs, whose offspring was used in the experiments. All experiments were undertaken in mice of 4–6 months of age. The animals were maintained in a temperature-controlled room (22°C) on a 12:12 h light-dark cycle (light on at 7:00 AM). Mice were fed a standard pellet diet (total energy 14.1 MJ/kg with 14% from fat, 60% from carbohydrate and 26% from protein; SAFE, Augy, France) and tap water ad libitum. During experimental days, food was removed from the cages at 7:30 AM and the actual experiments started at 12:30, i.e., during the light cycle. We used only female mice to avoid the stress of single housing, which is used in male mice, and to be in line with the previous studies on GIP receptor and GLP-1 receptor knockout mice (20). We used the mice randomly during the estrous cycle. The study was approved by the Lund/Malmö Animal Ethics Committee (Approval No. 5.8.18-06417/2020) and performed according to Good Laboratory Practice.

A total of 48 animals were assigned for the evaluation of the novel anesthesia formulation and a total of 99 animals were allocated for the experimental procedure to study insulin independent glucose disposal (52 wildtype mice, 23 GIP receptor knockout mice and 24 GLP-1 receptor knockout mice). Studies were undertaken in groups of 6–8 mice on each experimental day by one experienced technician. In all individual experiments, animals from all individual subgroups were involved to avoid bias in different results on different days. All individual results from the completer population were included in the final analysis and statistics.

### Anesthesia

Mice were anesthetized with the neurolept anesthesia using the combination of fentanyl citrate, fluanisone and midazolam. A novel mixture of fentanyl citrate/fluanisone was used (Fluafent) and this was initially validated versus the conventional fentanyl citrate/fluanisone combination (Hypnorm^R^) which has been used in previous work (6, 8–10, 15, 20, 21). The conventional Hypnorm^R^ anesthesia was instituted after intraperitoneal administration of 100µl of a fixed dose combination of fentanyl citrate (0.02 mg/mouse)-fluanisone (0.5 mg/mouse) (Hypnorm^R^; Vetpharma, Leeds, UK). In the novel anesthesia, called Fluafent, 10 mg fluanisone (Key Organics, Camelford, Cornwall, UK) was dissolved in 1 ml sterile water at 78°C for 60 min. This solution was mixed with 1 ml of fentanyl citrate (Sigma-Aldrich, St Louis, MO; 0.315 mg/ml); 100µl of this solution were given intraperitoneally to each mouse (0.020 mg fentanyl citrate and 0.75 mg fluanisone/mouse). In both anesthesia procedures, midazolam (0.125 mg/mouse; Roche, Basel, Switzerland) was also given (100µl/mouse).

### Intravenous Glucose Test

In the experimental series with anesthesia validation, after a 5-h fast, wildtype mice were anesthetized with either of the two anesthetics. Mice were then given an intravenous bolus dose of D-glucose over 3 seconds (dissolved in saline; Sigma) in a tail vein at the dose of 0.35 g/kg. In the series examining insulin-independent glucose disposal, after a 5-h fast, wildtype, GIP receptor knockout and GLP-1 receptor knockout mice were anesthetized with the combination of Fluafent/midazolam as above. Mice were then given a subcutaneous administration of 25mg/kg diazoxide (dissolved in NaOH) (21) or vehicle, followed after 20 minutes by an intravenous bolus dose of D-glucose over 3 seconds (dissolved in saline; Sigma) in a tail vein at the dose of 0.35 g/kg. Whole blood was sampled in heparinized pipettes from the intraorbital retrobulbar sinus plexus (40 µl) immediately before glucose injection (time t=0) and at 1, 5, 10, 20 and 50 min after glucose injection. Glucose was measured in the blood samples, and then plasma was separated by centrifugation and stored at -20°C until analysis for insulin.

### Oral Glucose Test

After a 5-h fast, wildtype mice were anesthetized with either of the two anesthetics as above and a gastric tube (outer diameter 1.2 mm) was placed in the stomach. A blood sample was taken as above, then glucose (75 mg in volume of 0.3ml) was administered in the tube and blood was taken at 15, 30, 45 and 60 min after glucose administration. Glucose was measured in the blood samples, and then plasma was separated by centrifugation and stored at -20°C until analysis for insulin.

### Assays

Glucose was analyzed with the glucose oxidase method using Accu Chek Aviva (Hoffman-La Roche, Basel, Switzerland). Insulin was determined by ELISA (Mercodia, Uppsala, Sweden). The intra-assay coefficient of variation (CV) of the method is 4% at both low and high levels, and the interassay CV is 5% at both low and high levels. The lower limit of quantification of the assay is 6 pmol/l.

### Data Analysis

Glucose disappearance rate was estimated as the net glucose elimination rate after the glucose injection (K_G_, the glucose tolerance index) as the slope from the peak glucose for the following 5–20 min after glucose injection of the logarithmic transformation of the individual plasma glucose values (8). Insulin sensitivity index (S_I_) and glucose effectiveness (S_G_) were evaluated with the minimal model technique as extensively explained elsewhere (9, 10, 23, 24). S_I_ is defined as the ability of insulin to enhance net glucose disappearance and inhibit glucose production, whereas S_G_ is the net glucose disappearance per se from plasma without any change in dynamic insulin. Suprabasal area under the curves for glucose (AUC_glucose_) and insulin levels (AUC_insulin_) were calculated by the trapezoid rule and beta-cell glucose sensitivity was estimated by AUC_insulin_ divided by AUC_glucose_. Data and results are shown as mean ± SEM.

### Statistical Analysis

Differences between experimental groups were determined using Student’s unpaired *t*-test by comparing data obtained in GIP receptor knockout and GLP-1 receptor knockout mice, respectively, with their respective wildtype controls included in the same experimental groups, and by comparing data obtained after the two anesthetic formulations, respectively. Insulin levels were handled statistically after logarithmic transformation since plasma insulin levels in mice are not normally distributed, whereas glucose levels are normally distributed (20). For all analyses, statistical significance was defined as *P <*0.05. Analyses were carried out using SPSS, v. 27.

## Results

### Validation of Fluafent in Comparison With Hypnorm^R^

**Figure 1:**
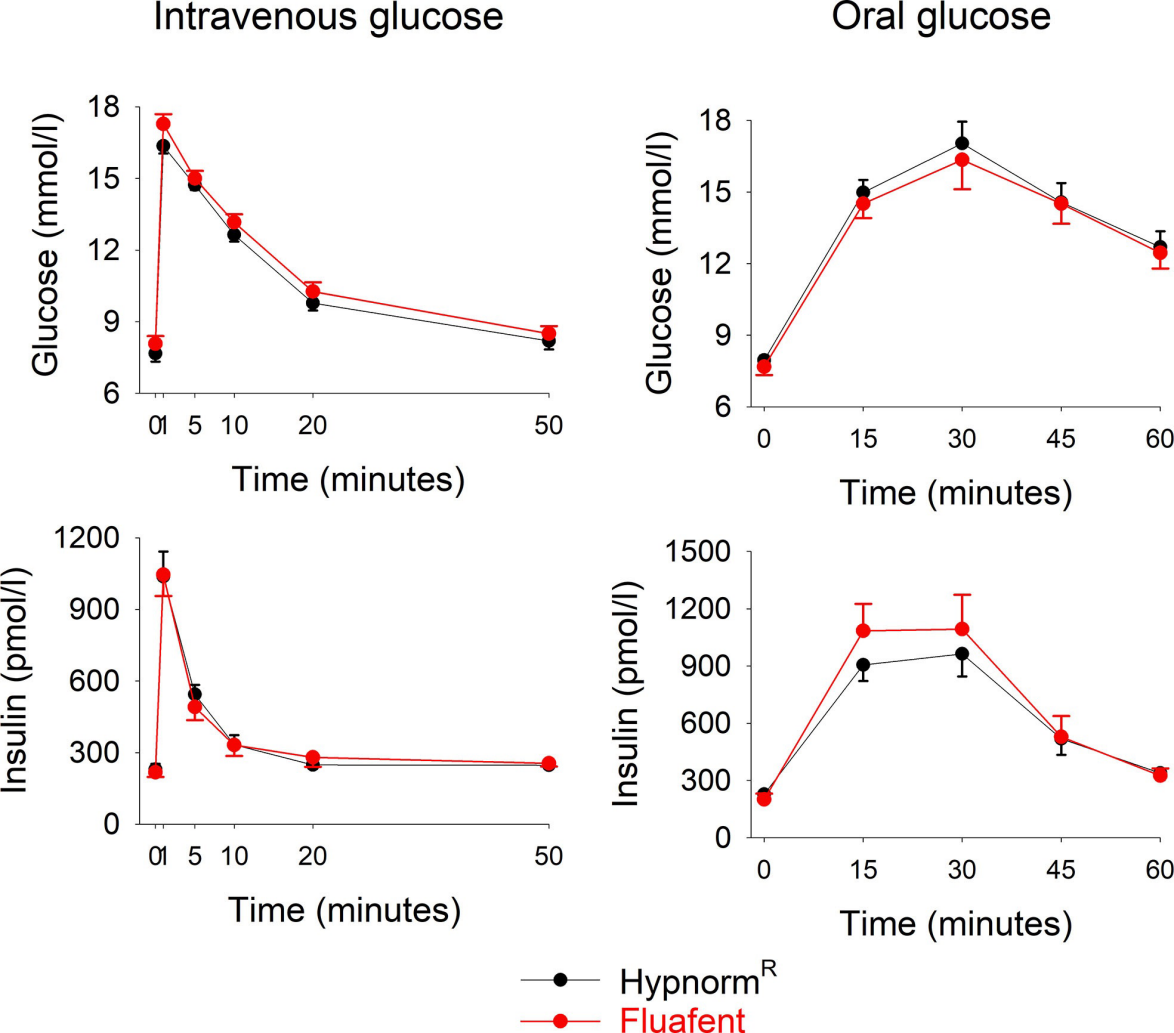
Glucose and insulin levels after intravenous injection of glucose (0.35g/kg; left panels) or after oral administration of glucose (75 mg; right panels) in C57BL/6J mice during anesthesia with Hypnorm^R^/midazolam or Fluafent/midazolam. There were 12 animals in each of the four study groups. Means ± SEM are shown.

Wildtype mice were anesthetized with neuroleptic anesthesia using the combination of fentanyl citrate and fluanisone either as Fluafent or as Hypnorm^R^, both in combination with midazolam. Baseline glucose and insulin levels did not differ between the two anesthetics. After intravenous glucose administration, glucose and insulin levels increased with peaks at 1 min (**Figure 1**). There was no significant difference between the two anesthetics in any of the parameters at any of the time points. Also in the experimental series with oral glucose, baseline glucose and insulin levels did not differ between the two anesthetics. After oral glucose administration, glucose and insulin levels increased with peaks at 15 min followed by a gradual return towards baseline values (**Figure 1**). Again, there was no difference between the two anesthetics in these data.

### Insulin-Independent Glucose Disposal

**Figure 2:**
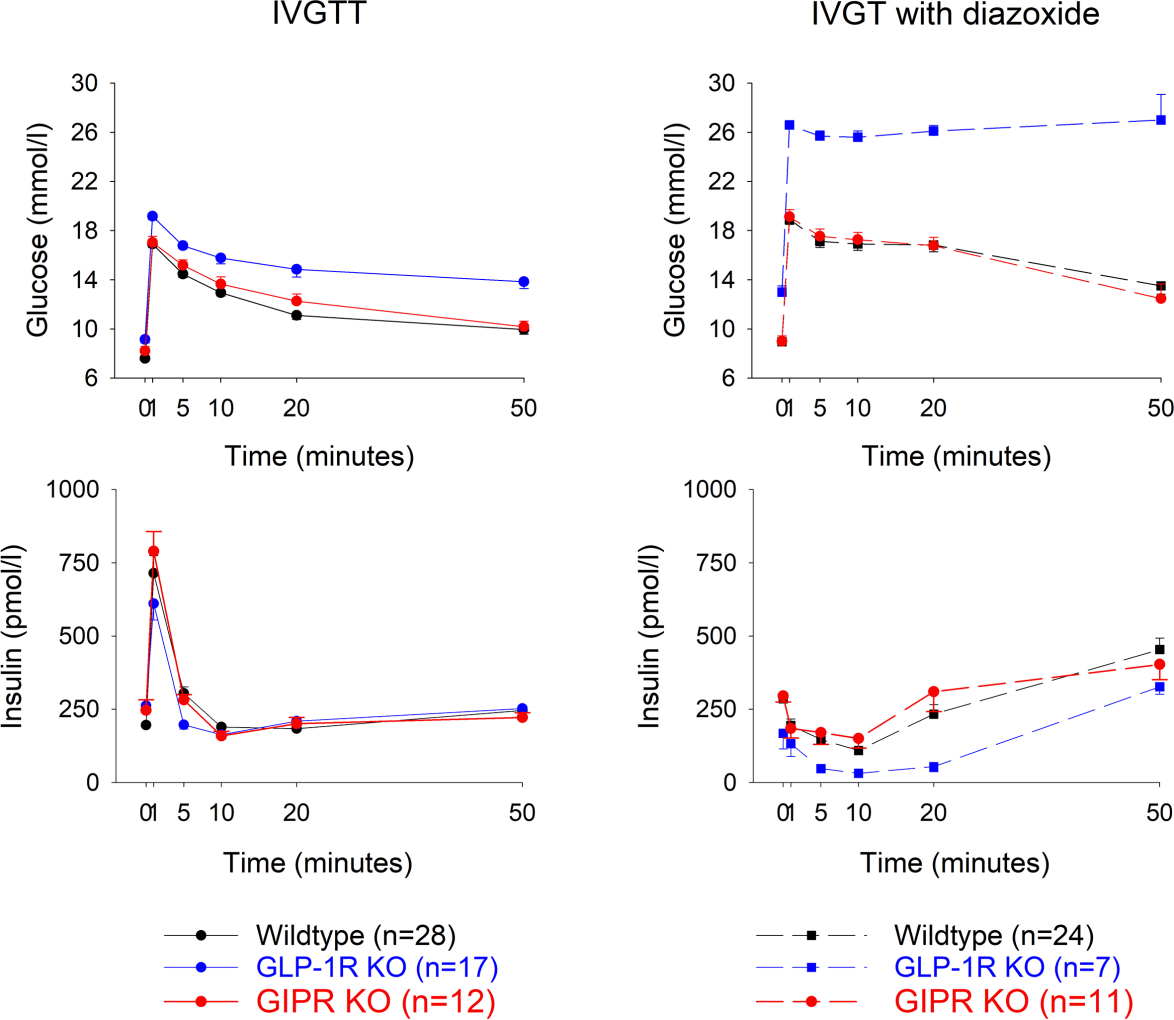
Glucose and insulin levels after intravenous injection of glucose (0.35g/kg) with a subcutaneous administration of a vehicle (left panels) or diazoxide (25 mg/kg, right panels) in wildtype, GLP-1 receptor knockout or GIP receptor knockout mice. n indicates number of animals. Means ± SEM are shown.

Glucose effectiveness (S_G_) after IVGTT (**Figure 2** left panel). After intravenous glucose administration, glucose and insulin levels peaked after 1 min followed by a gradual reduction in all three study groups. Glucose elimination rate between mins 5 and 20 (K_G_) was 1.78 ± 0.14%/min in wildtype mice (n=28), 1.43 ± 0.18 in GIP receptor knockout mice (n=17) and 0.96 ± 0.18 in GLP-1 receptor knockout mice (n=12). Glucose effectiveness (S_G_) was 0.0646 ± 0.005 min^-1^ in wildtype, 0.0521 ± 0.005 in GIP receptor knockout mice and 0.037 ± 0.006 in GLP-1 receptor knockout mice. **Table 1** shows the statistical evaluation between animals examined in the same experiments. It is seen that K_G_ and S_G_ were significantly lower in GLP-1 receptor knockout mice than in wildtype mice, whereas K_G_ and S_G_ were not significantly different between GIP receptor knockout and wildtype mice. Furthermore, suprabasal AUC_insulin_ and beta-cell sensitivity were significantly lower in GLP-1 receptor knockout mice than in wildtype mice but not significantly different between GIP receptor knockout and wildtype mice. Finally, insulin sensitivity index (S_I_) was significantly lower in GIP receptor knockout mice than in wildtype mice, with no significant difference between GLP-1 receptor knockout and wildtype mice.

**Table 1:** Baseline glucose and insulin, suprabasal area under the 50 min curve (AUC) of glucose and insulin, glucose elimination rate (K_G_), glucose effectiveness (S_G_), insulin sensitivity index (S_I_) and beta-cell glucose sensitivity in vehicle-treated GIP and GLP-1 receptor knockout (KO) and their respective control wildtype mice.

	Wildtype	P	GIP receptor KO	Wildtype	P	GLP-1 receptor KO
n	11		12	10		17
Baseline glucose (mmol/l)	7.9 ± 0.3	0.49	8.2 ± 0.4	7.5 ± 0.4	0.005	9.1 ± 0.3
Baseline insulin (pmol/l)	183 ± 10	0.11	246 ± 36	178 ± 14	0.003	262 ± 21
Suprabasal AUC_glucose_ (mmol/l min)	159 ± 10	0.66	151 ± 13	155 ± 14	0.042	193 ± 10
Suprabasal AUC_insulin_ (nmol/l min)	1.12 ± 0.22	0.25	-41 ± 0.92	1.85 ± 0.43	0.001	-0.90 ± 0.62
K_G_ (%/min)	1.74 ± 0.25	0.34	1.43 ± 0.19	1.6 ± 0.2	0.042	1.0 ± 0.2
S_G_ (min^-1^)	0.060 ± 0.010	0.46	0.052 ± 0.005	0.060 ± 0.007	0.023	0.037 ± 0.006
S_I_ (10^-4^ min^-1^/(pmol)	1.45 ± 0.11	0.035	1.11 ± 0.10	1.23 ± 0.14	0.12	0.94 ± 0.12
Beta-cell sensitivity (mmol/mol)	0.017 ± 0.001	0.31	0.019 ± 0.001	0.019 ± 0.001	0.042	0.015 ± 0.001

Means ± SEM are shown. P indicates the probability level of random difference between the groups.

Glucose elimination after IVGTT with diazoxide (**Figure 2** right panels). Diazoxide was given before the intravenous glucose which abolished the early insulin response. This was followed by increased baseline glucose and reduced suprabasal AUC_insulin_, K_G_, S_G_, S_I_ and beta-cell glucose sensitivity (compare **Table 1** *vs* **Table 2**). **Table 2** shows that also after diazoxide, as after vehicle treatment, S_G_ was significantly lower in GLP-1 receptor knockout mice than in wildtype mice with no significant difference between GIP receptor knockout mice and wildtype mice. In contrast, the markedly reduced K_G_ and S_I_ after diazoxide were not reduced further in GIP receptor knockout mice or in GLP-1 receptor knockout mice.

**Table 2:** Baseline glucose and insulin, suprabasal area under the 50 min curve (AUC) of glucose and insulin, glucose elimination rate (K_G_), glucose effectiveness (S_G_), insulin sensitivity index (S_I_) and beta-cell glucose sensitivity in diazoxide-treated GIP and GLP-1 receptor knockout (KO) and their respective control wildtype mice.

	Wildtype	P	GIP receptor KO	Wildtype	P	GLP-1 receptor KO
n	11		11	6		7
Baseline glucose (mmol/l)	9.5 ± 0.5	0.43	9.0 ± 0.4	8.2 ± 0.4	<0.001	13.0 ± 0.5
Baseline insulin (pmol/l)	302 ± 33	0.85	295 ± 20	306 ± 57	0.11	168 ± 54
Suprabasal AUC_insulin_ (nmol/l min)	-3.43 ± 0.75	0.14	-1.56 ± 0.95	-3.93 ± 1.68	0.44	-2.03 ± 1.68
K_G_ (%/min)	0.35 ± 0.12	0.034	0.79 ± 0.15	0.45 ± 0.09	0.63	0.37 ± 0.12
S_G_ (min^-1^)	0.012 ± 0.003	0.38	0.016 ± 0.003	0.011 ± 0.026	0.019	0.003 ± 0.001
S_I_ (10^-4^ min^-1^/(pmol)	0.26 ± 0.04	0.12	0.48 ± 0.13	0.19 ± 0.03	0.13	0.44 ± 0.14
Beta-cell sensitivity (mmol/mol)	0.011 ± 0.001	0.21	0.015 ± 0.002	0.011 ± 0.02	0.016	0.004 ± 0.001

Means ± SEM are shown. P indicates the probability level of random difference between the groups.

## Discussion

As recently reviewed, insulin-independent glucose disposal is an important component for glucose elimination (5, 6). It has also been shown that insulin-independent glucose disposal is impaired in obesity and type 2 diabetes (11–13). These findings together support the conclusion that insulin-independent mechanisms are important for glucose disposal and may be of relevance for diabetes pathophysiology. Previous studies have shown that exogenous administration of GLP-1 increases S_G_ in model experiments in mice (6, 15, 21) and in humans (14). This would suggest that the glucose-lowering ability of GLP-1 is mediated by both insulin-dependent (through increase in insulin levels) and insulin-independent mechanisms. In the present study, we examined if removal of GLP-1 action through genetic deletion of GLP-1 receptors in mice affect glucose effectiveness, i.e., if an action of GLP-1 is required for normal insulin-independent glucose disposal. We used two approaches for the studie. First, we estimated S_G_ after a normal intravenous glucose injection allowing insulin levels to change markedly. Second, we also estimated S_G_ after diazoxide, which is a drug that completely inhibits insulin secretion (19). We show with both these approaches that S_G_ is reduced in GLP-1 receptor knockout mice, which confirms an earlier study on S_G_ after intravenous glucose in mice (20). This indeed suggests that insulin-independent gluose disposal is not only activated by administration of GLP-1 but also dependent on active GLP-1 receptors for full operation.

The mechanism of insulin-independent glucose disposal is not established but it has been discussed that it is exerted in the liver and in muscles (6). Experimental support for this is that S_G_ is reduced in cirrhotic patients (25) and an experimental study on non-insulin-mediated glucose disposal in humans shows its action in muscles (26). Since GLP-1 has been demonstrated to reduce glucose release from the liver both in humans and animals (27, 28), it is possible that GLP-1 mediates insulin-independent glucose disposal through these effects.

In contrast to the impairment of insulin-independent glucose disposal in GLP-1 receptor knockout mice, our results in GIP receptor knockout mice showed no difference from wildtype mice, both under normal conditions and after diazoxide. This suggests that active GIP receptors are not required for a normal insulin-independent glucose disposal. The two incretin hormones therefore show dissociated effects in this respect. This is different from the increase in S_G_ which is evident after exogenous administration not only of GLP-1 but also of GIP in mice (15).

We thus found that GLP-1 seems to be an important contributor to insulin-independent glucose disposal in mice. Important aspects of this is whether this is the same in humans and to what extent GLP-1 contributes to the process. The regulation of insulin-independent glucose disposal in humans was recently reviewed (6) and in comparison to mice much similarities exist. In regard to incretin hormones, GLP-1 increases insulin-independent glucose disposal in both man and mice (14, 15) whereas a potential effect of GIP has not been examined in humans yet. The potential relevance of GLP-1 receptors for insulin-independent glucose disposal in man, as demonstrated in the present study in mice, remains also to be studied. In regard to the quantitative contribution by GLP-1 of insulin-independent glucose disposal, we found in the present study that S_G_ was reduced by ≈43% in GLP-1 receptor knockout mice compared to wildtype mice. This suggests that GLP-1 contributes to a large extent to insulin-independent glucose disposal. This is supported by our previous study which showed that exogenous administration of GLP-1 increases S_G_ by ≈35%, which also suggests a large contribution by GLP-1 (15). There are also other factors that potentially contribute to the regulation of insulin-independent glucose disposal, such as insulin sensitivity and glucagon, and this was recently reviewed (6).

This study was the first project in which the novel neurolept anesthesia formulation Fluafent was used. Neurolept anesthesia has been a commonly used anesthetic procedure in humans since it was introduced clinically more than 60 years ago (29, 30) and has become a common anesthetic procedure in animals (31). An often used neurolept anesthesia is the combination of fentanyl, fluanisone and midazolam. Fentanyl is a synthetic analgesic with a higher potency than morphine and is characterized by rapid onset and short duration of action, while fluanisone is an antipsychotic sedative belonging to butyrophenone group which augments the analgesic action of fentanyl (31). Midazolam belongs to benzodiazepine drugs which induce sleepiness and reduce anxiety (32). This anesthesia has been shown to be a preferred anesthesia in experimental animal studies in inflammatory, hemodynamic and metabolic research (23, 33–35).

The combination of fentanyl citrate/fluanisone has been offered to animal scientists as Hypnorm^R^. However, Hypnorm^R^ is frequently difficult to obtain, which has been a great challenge for experimental researchers during recent years. There is therefore a need for an alternating fentanyl citrate/fluanisone combination which is as reliable as Hypnorm^R^. We have developed such a combination, which we call Fluafent. Here we have reported the first study using this novel anesthesia formulation. We show that glucose and insulin data after intravenous and oral glucose in mice were not significantly different after Fluafent/midazolam anesthesia compared to Hypnorm^R^/midazolam anesthesia. Therefore, Fluafent offers a reliable replacement for Hypnorm^R^.

In conclusion, we show in this study that the novel neurolept anesthesia Fluafent/midazolam is comparable to Hypnorm^R^/midazolam in regard to glucose and insulin data after intravenous and oral glucose in mice, and that incretin hormone receptors show dissociated effects on insulin-dependent glucose elimination in mice, in that the process requires intact GLP-1 receptors, whereas in contrast intact GIP receptors is not crucial for this process.

## Data Availability Statement

The raw data supporting the conclusions of this article will be made available by the authors, upon reasonable request.

## Ethics Statement

The animal study was reviewed and approved by Lund/Malmö Animal Ethical Committee.

## Author Contributions

TO: Conceptualization, Data curation, Investigation, Methodology, Validation, Writing - review & editing. GP: Data curation, Formal analysis, Methodology, Software, Validation, Writing - review & editing. BA: Conceptualization, Data curation, Formal analysis, Funding acquisition, Investigation, Methodology, Project administration, Resources, Software, Supervision, Validation, Writing - original draft, Writing - review & editing. All authors contributed to the article and approved the submitted version.

## Funding

This research was funded by the Lund University Medical Faculty, Region Skåne, and the Swedish Research Council (to BA).

## Conflict of Interest

The authors declare that the research was conducted in the absence of any commercial or financial relationships that could be construed as a potential conflict of interest.
